# Identifying critically ill patients who benefit the most from nutrition therapy: the development and initial validation of a novel risk assessment tool

**DOI:** 10.1186/cc10546

**Published:** 2011-11-15

**Authors:** Daren K Heyland, Rupinder Dhaliwal, Xuran Jiang, Andrew G Day

**Affiliations:** 1 Clinical Evaluation Research Unit, Kingston General Hospital, Kingston, ON, Canada; 2 Department of Community Health and Epidemiology, Queen's University, Kingston, ON, Canada; 3 Department of Medicine, Queen's University, Kingston, ON, Canada

## Abstract

**Introduction:**

To develop a scoring method for quantifying nutrition risk in the intensive care unit (ICU).

**Methods:**

A prospective, observational study of patients expected to stay > 24 hours. We collected data for key variables considered for inclusion in the score which included: age, baseline APACHE II, baseline SOFA score, number of comorbidities, days from hospital admission to ICU admission, Body Mass Index (BMI) < 20, estimated % oral intake in the week prior, weight loss in the last 3 months and serum interleukin-6 (IL-6), procalcitonin (PCT), and C-reactive protein (CRP) levels. Approximate quintiles of each variable were assigned points based on the strength of their association with 28 day mortality.

**Results:**

A total of 597 patients were enrolled in this study. Based on the statistical significance in the multivariable model, the final score used all candidate variables except BMI, CRP, PCT, estimated percentage oral intake and weight loss. As the score increased, so did mortality rate and duration of mechanical ventilation. Logistic regression demonstrated that nutritional adequacy modifies the association between the score and 28 day mortality (p = 0.01).

**Conclusions:**

This scoring algorithm may be helpful in identifying critically ill patients most likely to benefit from aggressive nutrition therapy.

## Introduction

Identifying patients who are at risk of adverse events because of their nutrition status is a core competency of nutrition practitioners, recommended by clinical practice guidelines, and mandated by accreditation agencies [1-3]. Inherent in this discussion of nutrition risk is that patients at high risk are more likely to benefit from nutritional therapeutic interventions than those at low risk, as nicely demonstrated by Kondrup and colleagues [4]. Many scores or assessment tools exist to enable the quantification of nutrition risk [5-10]. For the most part, these tools were developed and validated in outpatient or inpatient settings but not specifically for the ICU setting [11]. In fact, most scores consider that all critically ill patients are at a high risk in terms of their scoring or risk assessment [4,5].

We posit that this is not the case, and that not all critically ill patients are the same in terms of their nutritional risk. The evidence for this assertion comes from studies that demonstrate a differential treatment effect of artificial nutrition in different subgroups of ICU patients. In a recent analysis, we observed a significant inverse linear relation between the odds of mortality and total daily calories received [12]. An increase of 1,000 calories per day was associated with an overall reduction in mortality (odds ratio for 60 day mortality 0.76, 95% confidence intervals (CI), 0.61-0.95, *P *= 0.014). However, the beneficial treatment effect of increased calories on mortality was observed in patients with a body mass index (BMI) below 25 or 35 and above with no benefit for patients with a BMI of between 25 or less than 35. Similar results were obtained when comparing increasing protein intake and its effect on mortality in different BMI groups. One of the main inferences from this work is that not all ICU patients are the same with respect to their response to artificial nutrition.

**Figure 1:**
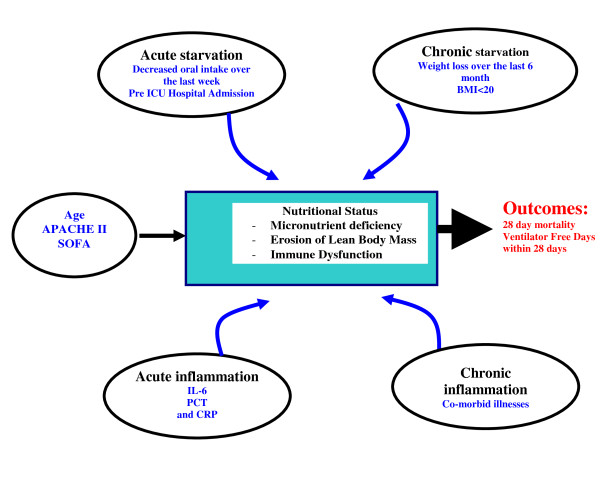
**Conceptual model for nutrition risk assessment in the critically ill**. APACHE, Acute physiology and chronic health evaluation score; BMI, body mass index; CRP, C-reactive protein; IL-6, interleukin 6; PCT, procalcitonin; SOFA, sequential organ failure assessment score.

So how do we begin to approach discriminating 'nutritional risk' in the critical care setting? In a recent International Consensus Guideline statement, Jensen and colleagues offer some ground breaking definitions of malnutrition relating it to both acute and chronic malnutrition and inflammation [13]. Consistent with this definition, in Figure 1, we present our conceptual model of how measures of acute and chronic starvation and inflammation may influence nutrition status at ICU admission and ultimately impact on patient outcomes. Our ultimate goal was to develop a score using the variables presented in the model that would quantify the risk of an individual patient developing adverse events and that may be potentially modifiable by aggressive or adequate nutritional intervention. In fact, to validate our score, we not only had to demonstrate that it discriminated risk among a heterogenous group of ICU patients, but also that the association between the risk score and outcome was modified by nutritional invention.

Thus, the purpose of the study was to develop and validate a novel method for quantifying risk of adverse outcomes that may be modified by nutrition therapy in the critical care setting, the NUTrition Risk in the Critically ill (NUTRIC score).

## Materials and methods

### Patients and setting

This study was a secondary analysis of a prospective observational study in three tertiary care, surgical-medical ICUs conducted to evaluate a novel diagnostic marker for sepsis [14]. Patients at least 18 years of age were enrolled within 24 hours of admission to the ICU. Patients admitted for elective surgery, those admitted with overdoses, and patients who were expected to stay less than 24 hours were excluded. Given the nature of this observational study, no attempt was made to standardize care, including nutritional practices, across participating ICUs. The clinical management of patients was determined by the clinical team looking after the patient and the clinical protocols operational in participating ICUs at that time. Patients were started on enteral nutrition within 24 to 48 hours of ICU admission (on average), according to local practice. Feeds were advanced or continued at goal hourly rate if the gastric residuals (checked every four hours) were less than 200 to 250 ml. The local dietitians determined the goal rates using standard formulae. Gastrointestinal prokinetic agents and, eventually, small bowel feeding tubes were prescribed in the event of problems with persistent high gastric residual volumes. When clinically indicated, parenteral nutrition was prescribed by the clinical team. Arterial or venous blood glucose levels were assessed daily in the morning and frequently through the day and a glycemic control protocol was used to prescribe the dose of insulin to titrate blood sugars between 4.0 and 9.0 mmol/L.

This study was approved by the Queen's Research Ethics Board and informed consent was obtained from all participating patients or their substitute decision-makers.

### Clinical data collection

All data were collected prospectively. Research coordinators interviewed family members, where available, to obtain historical nutrition variables (recent reduction in intake by mouth (% in last week) and history of weight loss in the past six months). Data on baseline demographics, past medical history including a detailed list of comorbidities and medications were abstracted from patients' charts. Acute Physiology and Chronic Health Evaluation Scores (APACHE II) [15] and Sequential Organ Failure Assessment (SOFA) scores [16] variables were recorded on admission to ICU. Data pertaining to nutrition prescriptions and intake were collected daily until death or discharge from the ICU, or to a maximum of 14 days. Percentage adequacy of nutrition was calculated as energy or protein actually received divided by total energy or protein prescribed. Outcomes were collected until day 28 and these included ventilator free days in 28 days; ICU length of stay, and 28-day mortality.

### Laboratory measurements

Upon enrollment and daily thereafter until ICU discharge, death or a maximum of 10 days, morning blood samples were collected. Plasma was analyzed for inflammatory markers using the following assays: C-reactive protein (CRP) by the CRPh reagent Beckman Coulter Unicel DxC 600/800 Synchron Clinical System (Hoffman - La Roche Ltd, Basel, Switzerland), procalcitonin (PCT) using the BRAHMS PCT LIA, B·R·A·H·M·S (Berlin, Germany); IL-6 using the Bender MedSystems ELISA Kit-Cat. BMS213 (Bender MedSystems Inc, Burlingame, CA, USA).

### The conceptual model

To develop this nutrition risk score, we first started with a conceptual model that linked starvation, inflammation, nutrition status, and clinical outcomes (Figure 1). Potential variables to represent these constructs were chosen based on their fit with the conceptual model and their ease of routine use. With respect to starvation and inflammation, we considered that there would be two forms, both acute and chronic. We considered recent decreased oral intake [4-9] and pre-ICU stay in hospital [17] as candidate variables for acute starvation and a history of recent weight loss [4-9] (within past three months) and a low BMI (current BMI < 20) [4,6,7] as measures for chronic starvation. To represent inflammatory markers, we were limited by the measurements available to us from the original study. We chose PCT, IL-6, and CRP to be representative markers of acute inflammation and the presence of comorbid illnesses to reflect a measure of chronic inflammation. All of the variables selected based on the conceptual model were candidates for the inclusion in the NUTRIC score algorithm. We expected this model to explain additional mortality risk, above and beyond what would be derived from use of traditional measures of severity of illness (APACHE II score and baseline SOFA).

### Statistical approach

Our first step was to validate our choice of candidate variables, derived from our conceptual model, by describing their association with 28-day outcomes. Candidate variables were compared between 28-day survivors and non-survivors. Categorical variables were described as counts and percentages and compared by the Chi-Square test whereas continuous variables were described as medians and inter-quartile range (IQR) and compared by the Wilcoxon rank-sum test. The Spearman correlation coefficient was used to assess the association between patient characteristics and candidate variables and ventilator-free days within 28 days.

In our second step, we developed the NUTRIC score using the candidate predictor variables. Percent oral intake in the week prior to enrolment was dichotomized into patients who reported less than 100% versus everyone else including those without this variable reported. This dichotomization did not result in substantial information loss because only 10% of patients report less than 100% but more than 10% oral intake. Similarly, as 76% of patients reported less than 1% weight loss, weight loss was dichotomized as patients who reported any weight loss versus everyone else. A sensitivity analysis was performed where we assumed that weight loss or less than 100% oral intake did occur when not reported. BMI was dichotomized as less than 20 versus other as the data were too sparse to have multiple BMI categories. The number of comorbidities was left as integer values (range 0 to 5). The remaining candidate variables were categorized into five equal sized groups (quintiles). The categorized candidate variables were then each fit as categorical predictors in separate single predictor logistic regression models predicting 28-day mortality. The parameters for each logistic regression model estimate the log of the odds ratio (logit) for each category (usually quintile) of the variable compared with the lowest risk (reference) category. These parameters were rounded to whole numbers to provide the points used in the NUTRIC risk score. Equal point categories were collapsed, and the exact quintile ranges were subsequently rounded to convenient values. The total NUTRIC score was simply the sum of the points across all included variables. Variables with an overall significance of more than 0.2 or with all categories assigned 0 points were excluded from the scoring algorithm. Furthermore, variables were excluded if their inclusion in the NUTRIC score did not improve the score's ability to predict 28-day mortality. The resulting total scores ranged from 0 (lowest risk category for all included variables) to 10 (highest risk category for all included variables). We choose this simple approach to model building over more sophisticated and data-dependent methods because it was intuitive and (given our relatively limited sample size) less susceptible to overfitting and optimism bias [18,19]. Nevertheless, we did confirm that the multivariable fractional polynomial approach proposed by Saurerbrei and Royston yielded a similar model with no improvement in performance (data not shown) [20,21].

In step 3 we evaluated the quality of the NUTRIC score model for predicting 28-day mortality. Model discrimination was assessed by the C-statistic derived from calculating the area under the receiving operating characteristic curve (interpretation of c-index: excellent ≥0.90, adequate 0.70 to 0.89, poor < 0.70) and the generalized max-rescaled R-squared statistic [18,22]. These statistics were also used to compare the discriminative capacity of the NUTRIC score model with logistic model with only age, APACHE II score and baseline SOFA and logistic model excluding any measure of acute inflammation. Model calibration (i.e. goodness of fit) was assessed descriptively by visually comparing the predicted (model based) and actual (observed) mortality rates for each score value, and formally by the Hosmer-Lemeshow goodness of fit test [23]. The performance of the scoring algorithm was cross-validated by independently deriving the NUTRIC score using a random split half of the sample and then evaluating its discriminative ability on the other half sample.

To further validate the NUTRIC score, we examined the association between the NUTRIC score and mechanical ventilation (MV) duration among 28-day survivors. The agreement between observed and model-based estimates of MV duration for each NUTRIC score value was also examined. Our *a priori *hypothesis was that patients with a higher NUTRIC score would have a longer duration of MV.

In our final step, we examined if the risk score modified the association between nutritional intake and 28-day mortality in a subset of patients who started MV within 48 hours after ICU admission and stayed in ICU for three days or longer (*n *= 211). *A priori*, we hypothesized that among patients who remained in ICU more than three days, those with high risk scores would benefit more from more nutritional intake whereas nutrition intake would not be as important in patients with low NUTRIC scores. Nutrition intake was expressed as the total amount of energy received from either enteral nutrition (EN) or parenteral nutrition (PN) over the number of ICU days divided by the amount prescribed as per the baseline assessment and expressed as percentage. Data from the last day in the ICU (unless day 14) were excluded in the calculation of the nutrition intake as these are only partial days on which we would not expect patients to receive their entire prescriptions. The association between nutritional intake and 28-day mortality was plotted by risk score. Logistic regression with nutritional intake, risk score and their product as continuous independent variables was used to generate a plot of the association between nutritional intake and 28-day mortality by risk score and to perform a likelihood ratio test for an interaction (effect modification) between NUTRIC score and nutritional intake among this subgroup of patients. However, for clarity the figure groups risk scores as 0 to 5 and 6 to 10.

## Results

The 597 patients enrolled in the original study were included in this analysis. However, we were only able to obtain data of recent oral intake and weight loss in 171 patients. Only 211 patients remained in the ICU for a minimum of three days and were evaluable for assessment of nutritional intake and its relation to outcome relative to the NUTRIC score. Table 1 provides the patients characteristics of the overall sample and the subgroup evaluable for nutritional intake and outcome analysis. Table 2 compares the candidate predictors by 28-day survival status. All of the candidate predictors except BMI, CRP, percentage oral intake in prior week, and percentage weight loss in past three months were significantly associated with 28-day mortality (all *P*< 0.001). Furthermore, all of the candidate predictors except BMI were significantly associated with lower ventilation-free days (Table 3).

**Table 1:** Patient characteristics

	All patients (*n *= 597) *	Patients evaluable for nutritional adequacy (*n *= 211)†
Age	63.9 (51.7 to 73.3)	65.0 (52.4 to 74.4)
Gender		
*Female *	250 (41.8%)	91 (43.1%)
*Male *	348 (58.2%)	120 (56.9%)
Race		
*Asian or Pacific Islander*	3 (0.5%)	1 (0.5%)
*Black*	51 (8.5%)	10 (4.7%)
*Hispanic*	1 (0.2%)	0 (0.0%)
*Native American*	4 (0.7%)	1 (0.5%)
*White*	539 (90.1%)	199 (94.3%)
Baseline Apache II score	21.0 (16.0 to 27.0]	23.0 (19.0 to 28.0)
Baseline SOFA score	7.0 (5.0 to 9.0]	7.0 (5.0 to 10.0)
# of days in hospital prior to ICU admission	0.4 (0.0 to 2.8]	0.5 (0.1 to 3.4)
BMI	26.5 (23.2 to 31.3]	26.8 (22.9 to 32.0)
Diabetes	136 (22.7%)	52 (24.6%)
Number of co-morbidities	3.0 (1.0 to 4.0]	3.0 (1.0 to 4.0)
Admission category		
*Medical *	375 (62.7%)	164 (77.7%)
*Surgical*	222 (37.1%)	47 (22.3%)
Primary admission diagnosis		
*Cardiovascular/vascular *	51 (8.5%)	20 (9.5%)
*Respiratory *	166 (27.8%)	89 (42.2%)
*Gastrointestinal *	99 (16.6%)	25 (11.8%)
*Neurologic *	35 (5.9%)	10 (4.7%)
*Sepsis *	40 (6.7%)	19 (9.0%)
*Trauma *	47 (7.9%)	17 (8.1%)
*Metabolic *	40 (6.7%)	11 (5.2%)
*Post-operative conditions *	75 (12.5%)	15 (7.1%)
*Renal *	26 (4.3%)	1 (0.5%)
*Orthopedic *	18 (3.0%)	4 (1.9%)

APACHE II, Acute Physiology and Chronic Health Evaluation; BMI, body mass index; SOFA, Sequential Organ Failure Assessment.

Values are median (q1 to q3) or n (%).

* These patients were used for developing and evaluating the NUTRIC score.

**† **The association between nutritional adequacy and mortality (by NUTRIC score) was assessed in the 211 patients who started mechanical ventilation within 48 hours after ICU admission, stayed in the ICU for three days or longer and recorded caloric intake.

**Table 2:** NUTRIC candidate variables by 28-day mortality status

	Non-survivors (*n *= 138)	Survivors (*n *= 460)	*P *values
Age	71.7 (60.8 to 77.2)	61.7 (49.7 to 71.5)	**< 0.001**
Baseline APACHE II score	26.0 (21.0 to 31.0)	20.0 (15.0 to 25.0)	**< 0.001**
Baseline SOFA	9.0 (6.0 to 11.0)	6.0 (4.0 to 8.5)	**< 0.001**
# of days in hospital prior to ICU admission	0.9 (0.1 to 4.5)	0.3 (0.0 to 2.2)	**< 0.001**
Baseline BMI	26.0 (22.6 to 29.9)	26.8 (23.4 to 31.5)	0.13
BMI			0.66
*< 20*	6 (4.3%)	25 (5.4%)	
*≥20*	122 (88.4%)	414 (90.0%)	
# of co-morbidities at baseline	3.0 (2.0 to 4.0)	3.0 (1.0 to 4.0)	**< 0.001**
Co-morbidity			**< 0.001**
*Patients with 0-1 co-morbidity*	20 (14.5%)	140 (30.5%)	
*Patients with 2 or more co-morbidities *	118 (85.5%)	319 (69.5%)	
C-reactive protein^¶^	135.0 (73.0 to 214.0)	108.0 (59.0 to 192.0)	0.07
Procalcitonin^¶^	4.1 (1.2 to 21.3)	1.0 (0.3 to 5.1)	**< 0.001**
Interleukin-6^¶^	158.4 (39.2 to 1034.4)	72.0 (30.2 to 189.9)	**< 0.001**
**171 patients had data of recent oral intake and weight loss**			
	**Non-survivors by day 28** **(*n *= 32)**	**Survivors by day 28** **(*n *= 139)**	***P *values**
% Oral intake in the week prior to enrolment	4.0 (1.0 to 70.0)	50.0 (1.0 to 100.0)	0.10
% of weight loss in the last three months	0.0 (0.0 to 2.5)	0.0 (0.0 to 0.0)	0.06

APACHE II, Acute Physiology and Chronic Health Evaluation; BMI, body mass index; SOFA, Sequential Organ Failure Assessment.

Values are median (q1 to q3) or n (%).

^¶ ^Baseline refers to the first available data

**Table 3:** Correlation between NUTRIC candidate variables and ventilator-free days within 28 days

Variable	Spearman correlation	*P *values	Number of observations
Age	0.1891	**< 0.0001**	598
Baseline APACHE II score	0.3914	**< 0.0001**	598
Baseline SOFA	0.3857	**< 0.0001**	594
% Oral intake (food) in the week prior to enrollment	0.1676	**0.0234**	183
number of days in hospital prior to ICU admission	0.1387	**0.0007**	598
% of weight loss in the last three months	0.1828	**0.0130**	184
Baseline BMI	0.0581	0.1671	567
# of co-morbidities at baseline	0.0832	**0.0420**	598
Baseline CRP	0.1539	**0.0002**	589
Baseline Procalcitonin	0.3189	**< 0.0001**	582
Baseline IL-6	0.2908	**< 0.0001**	581

APACHE II, Acute Physiology and Chronic Health Evaluation; CRP, C-reactive protein; SOFA, Sequential Organ Failure Assessment, ICU: Intensive Care Unit.

In Table 4 we present the variable ranges (i.e. collapsed and rounded quintiles) with their NUTRIC points used in the final scoring algorithm. BMI, CRP, PCT, weight loss, and oral intake were excluded because they were not significantly associated with mortality or their inclusion did not improve the fit of the final model. The final NUTRIC score was found to be predictive (c-index = 0.783) of 28-day mortality. For comparison, a logistic regression with only age, APACHE II score, and baseline SOFA score as continuous predictors had a c-index of 0.767 and a logistic regression model excluding any measure of acute inflammation (CRP, PCT, or IL-6) had a c-index of 0.776. The simple single predictor NUTRIC score model was as discriminative as the less parsimonious multiple logistic model with all six variables included in the NUTRIC score used as independent predictors (c-index = 0.781). The final model uses the overall data, but the two split sample results are provided to cross-validate the final model. It may be seen that the discriminative capacity remains strong and consistent across the two random split samples (c-index for sample A = 0.771 and for sample B = 0.770). Although there is some overlap between our candidate variables (e.g. APACHE II score includes age), co-linearity was not excessively high with the highest variance inflation factor reaching only 1.6 for APACHE II score. Furthermore, each variable remained independently statistically significant in the multivariable logistic model (*P*< 0.0001 for APACHE II score, *P *= 0.0016 for age and *P*< 0.1 for remaining variables).

**Table 4:** Proposed nutrition scoring system

	Overall (*n *= 598)		Random split A (*n *= 299)		Random split B (*n *= 299)	
Variables in NUTRIC Score	**Range**	**Points**	**Range**	**Points**	**Range**	**Points**

Age	< 50	0	< 50	0	< 60	0
	50-< 75	1	50-< 75	1	60-< 75	1
	≥75	2	75+	2	75+	2
APACHE II	< 15	0	< 15	0	< 15	0
	15-< 20	1	15-< 19	1	15-< 28	2
	20-28	2	19-28	2	28+	3
	≥28	3	28+	3		
SOFA	< 6	0	< 6	0	< 6	0
	6-< 10	1	6-< 10	1	6-< 10	1
	≥10	2	≥10	2	≥10	2
# Co-morbidities	0-1	0	0, 1	0		0
	2+	1	2, 3	1	1+	1
			4+	2		
Days from hospital to ICU admit	0-< 1	0	0<-1hr	0	ALL	0
	1+	1	1hr	1		
					220+	1
IL6	0-< 400	0	0-350	0	0-< 450	0
	400+	1	350+	1	450+	1
	
NUTRIC score discriminative performance	In sample		Out of sample		Out of sample	
	
AUC	0.783		0.771		0.770	
Gen R-Squared	0.169		0.163		0.157	
Gen Max-rescaled R-Squared	0.256		0.246		0.237	

APACHE II, Acute Physiology and Chronic Health Evaluation; AUC, area under the curve; SOFA, Sequential Organ Failure Assessment.

The proposed NUTRIC score is based on the overall sample. However, we randomly split the data into two halves to cross-validate its performance out of sample. The model developed by random split A was evaluated using random split B and vice versa.

**Figure 2:**
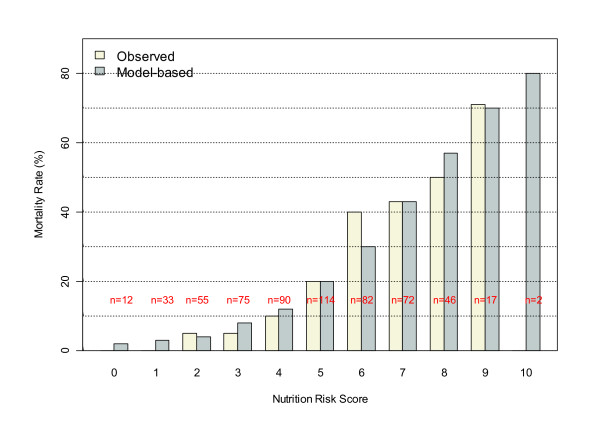
**Relation between NUTRIC score and 28-day mortality**. NUTRIC, Nutrition Risk in the Critically Ill Score.

**Figure 3:**
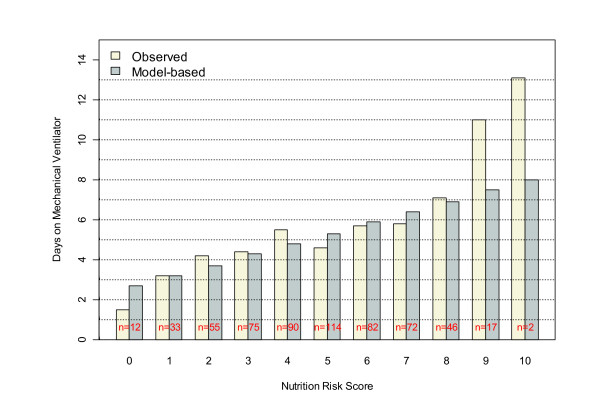
**Relation between NUTRIC score and duration of mechanical ventilation among 28-day survivors**. NUTRIC, Nutrition Risk in the Critically Ill Score.

Figure 2 displays the observed and logistic modeled mortality by NUTRIC score. It may be seen that mortality clearly increases with the NUTRIC score, and that the logistic model (with NUTRIC score as a continuous predictor) appears to adequately fit the observed data. Moreover, the Hosmer-Lemeshow test did not suggest a significant lack of fit (*P *= 0.28). Figure 3 shows that NUTRIC risk score is strongly associated with observed days on mechanical ventilation among 28-day survivors (*P *< 0.0001). Although it appears that the linear regression model somewhat attenuates this trend at the extreme ranges of the score, which may suggest a non-linear relation, it should be noted that there are few patients with NUTRIC scores of 0, 9, and 10 and the lack-of-fit test does not suggest the linear model is inadequate (*P *= 0.67).

**Figure 4:**
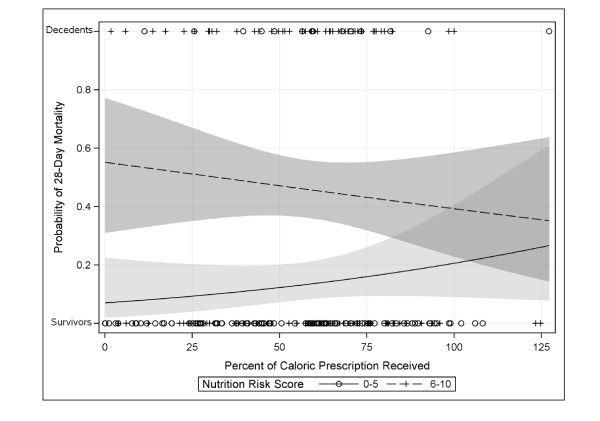
**Predicted probability of 28-day mortality versus percent of caloric**. Prescription received for patients with low (*n *= 114) and high (*n *= 97) NUTRIC score. Lines and shading are the predicted probability and 95% confidence intervals based on logistic regression. Circles and plus symbols indicate the observed values for the subgroup of 211 patients evaluable for assessment of nutritional adequacy. NUTRIC, Nutrition Risk in the Critically Ill Score.

Figure 4 visualizes the association between nutritional intake and mortality by risk score. It appears that the association between risk score and mortality is attenuated in patients who meet their caloric targets and that increased nutritional risk is associated with reduced mortality in high-risk patients only. This apparent effect modification is statistically significant (*P *= 0.01).

We found that patients with missing values for weight loss in the previous three months or percentage of oral intake in the prior week had similar elevated mortality rates as those that reported some weight loss or less than 100% oral intake. Grouping patients missing these variables with those reporting weight loss or less than 100% oral intake resulted in these variables being significantly associated with 28-day mortality. Nevertheless, the performance of the overall risk score did not improve (and in fact worsened slightly) by the inclusion of these re-defined nutrition variables.

## Discussion

Recognizing that not all ICU patients will respond the same to aggressive nutritional interventions, we set out to develop and conduct some preliminary validation work on a score that helps practitioners discriminate who might benefit the most (or least) from nutritional therapy. Borrowing heavily from Jensen and colleagues elaboration on the associations between nutritional state and inflammation [13], we developed a conceptual model that linked starvation, inflammation, and outcome. We chose candidate variables to represent acute and chronic starvation and inflammation and collected these data in a multicenter observational study of 597 patients. We demonstrated that, individually, these proposed variables did have a statistically significant relation with 28-day mortality and ventilator-free days, with the exception of BMI. These observations support the validity of our conceptual model linking measures of acute and chronic starvation and inflammation to outcome. BMI still may be an important determinant to outcome; however, we have very few patients with a low BMI (< 20) in this study. In other databases with larger numbers, BMI may still contribute to risk of adverse events and may be influenced by nutritional intake [12].

We reparameterized each variable and used regression techniques to determine the strength of the association between each derived variable and 28-day outcome. This process served to inform our scoring system. Unfortunately, we had difficulty in obtaining an accurate history of recent decreased oral intake and recent weight loss, a finding also noted in other studies [4,9,24], which seriously limits the clinical utility of these measurements. Despite the large amount of missing data, we attempted to include oral intake and recent weight loss by treating missing data both as normal and abnormal. Using this imperfect approach, these variables did not improve the fit of the regression models and were dropped from the score. However, it is possible that these variables would have come into the model had complete information been available. Further work with databases with more complete dietary history information may yield different results. Time in hospital prior to ICU stay was included in the NUTRIC score and can be considered as a marker for recent reduced oral intake as we know that iatrogenic malnutrition occurs commonly in hospitalized patients [25]. The various measures of acute inflammation did not increase the fit of the model by much either. CRP and PCT did not increase the fit of the model and were not included in the final score. IL-6 increased the c-index by only 0.007 points, which is neither clinically nor statistically different from the other scores. It was included in the final score because of the improvement in model fit but given the logistical problems and cost considerations in obtaining IL-6 levels in critically ill patients, we suggest that in settings where IL-6 is not readily available, it could be dropped from the score. There would be no added advantage to substituting CRP or PCT for IL-6 levels as the discriminative ability of the score did not improve with these variables included (data not shown). It should be acknowledged that the overall performance of the final model, as judged by the c-index, was still only 'adequate.' Further work needs to be conducted to enrich the signal and develop better scoring systems that enable us to determine which patients will benefit the most of aggressive nutrition therapy.

In the meantime, we think the NUTRIC score has some merit. In this paper, we have conducted several analyses to validate the derived scoring system and have demonstrated that patients with a higher score have worse clinical outcomes (high mortality and amongst survivors, longer duration of mechanical ventilation). We found that a logistic model with NUTRIC score as the sole continuous independent variable predicted mortality as well as a multivariable logistic model including all of the component variables. This substantial reduction in model degrees of freedom is especially important in small and medium size samples where multivariable models can be unstable and biased. Most importantly, in a subgroup of patients who stayed in ICU more than three days, we have demonstrated that patients with a high NUTRIC score benefit the most from aggressive provision of protein-energy requirements, towards meeting their estimated requirements. On the other hand, patients with a low score may even be harmed by such an approach. This adds strength to our prior observations that patients who receive more protein and energy have a better outcome [12] and further refines the observation by localizing the treatment effect in the sickest critically ill patients. Furthermore, this finding counters the argument that the sickest patients are the most difficult to feed and that is why we observe improve outcome in the better fed patient. In practical terms, the NUTRIC score (or some future derivative of it) may be used to help determine which patients receive supplemental parenteral nutrition or strategies to enhance EN delivery (such as motility agents, small bowel feeding tubes, and aggressive feeding protocols, such as the PEP uP protocol [26]).

The NUTRIC score, or the concepts contained therein, may have utility in the design and interpretation of clinical trials of nutrition therapies in the ICU setting. Studies that include heterogeneous ICU patients, some at high nutritional risk, some at low nutritional risk, are more likely to be negative than those who focus on treating only high-risk patients. Future clinical trials need to use the NUTRIC score or some other measurement tool validated in the ICU setting to describe 'nutrition risk' to enable an adequate interpretation of their findings.

One of the limitations of the NUTRIC score is that it only applies to the provision of macronutrients, protein, and energy. We do not expect this score to identify patients who may benefit more or less from pharmaconutrient supplementation (arginine, glutamine, antioxidants, for example). Likely, other biochemical measure of inflammation, immunity or nutrient values will have to provide clinicians with a sense of who will benefit the most from these specialized nutrients [27]. We further acknowledge that perhaps because the primary purpose of this study was to evaluate a sepsis marker, that standardization of nutrition practices and compliance with nutritional history variables was suboptimal. A major limitation of this dataset is that the nutrition history and intake information was only available in a minority of patients despite attempts to collect this data directly from families. Increased abstraction of these key nutrition variables in future studies may lead to further refinement of the NUTRIC score where these data variables may be included. Finally, it may be a limitation to the current derivation of the NUTRIC score that it is based on 28-day mortality, which was the only mortality data available to use in this dataset. Choosing longer term outcomes, such as 90-day mortality or some measure of functional status at hospital discharge may yield different but important results.

## Conclusion

We propose a novel scoring tool, the NUTRIC score, to help discriminate which ICU patients will benefit more (or less) from aggressive protein-energy provision. This scoring tool represents the first nutritional risk assessment tool developed and validated specifically for ICU patients. Whereas most methods of nutritional screening in hospitalized patients are reported to be cumbersome and time-consuming and hence are not routinely done [28], the NUTRIC score is a practical, easy-to-use tool based on variables that are easy to obtain in the critical care setting. The importance of this work rests not just in the statistical analyses presented, but at a conceptual level. We assert that not all ICU patients are the same, that there are some that benefit more (or less) from aggressive protein-energy provision in the critical care setting. Further refinement of this tool or others like it will ensure that the right ICU patient gets the right treatment and has implications for both clinical practice and designing future clinical trials.

## Key messages

• Not all ICU patients have the same risk for adverse consequences related to malnutrition.

• ICU-specific measurements of nutritional risk and validated in critically care settings are needed.

• Consideration of markers of acute and chronic inflammation and starvation can be used to guide the development of ICU specific measures of nutritional risk.

• The NUTRIC score is a valid scoring system that may be helpful in identifying critically ill patients most likely to benefit from aggressive nutrition therapy.

• Future clinical trials need to use the NUTRIC score or some other measurement tool validated in the ICU setting to describe 'nutrition risk' to enable an adequate interpretation of their findings.

## Abbreviations

APACHE II: Acute Physiology and Chronic Health Evaluation Scores; BMI: body mass index; CI: confidence interval; CRP: C-reactive protein; EN: enteral nutrition; IL-6: interleukin-6; IQR: interquartile range; MV: mechanical ventilation; NUTRIC: Nutrition Risk in the Critically Ill Score; PCT: procalcitonin; PN: parenteral nutrition; SOFA: Sequential Organ Failure Assessment.

## Competing interests

The authors declare that they have no competing interests.

## Authors' contributions

All authors read and approved the final manuscript. AD and XJ are responsible for the stats. DKH is responsible for the data collection.
